# GSTPi-positive tumour microenvironment-associated fibroblasts are significantly associated with GSTPi-negative cancer cells in paired cases of primary invasive breast cancer and axillary lymph node metastases

**DOI:** 10.1038/bjc.2011.352

**Published:** 2011-09-06

**Authors:** B Chaiwun, N Sukhamwang, H Trakultivakorn, B Saha, L Young, D Tsao-Wei, W Y Naritoku, S Groshen, C R Taylor, S A Imam

**Affiliations:** 1 Department of Pathology, Chiang Mai University, Chiang Mai, Thailand; 2 Department of Surgery, Chiang Mai University, Chiang Mai, Thailand; 3 Molecular Pathology Program, Huntington Medical Research Institutes, 99 N El Molino Avenue, Pasadena, CA 91101, USA; 4 Department of Pathology, USC Keck School of Medicine, Los Angeles, CA, USA; 5 Department of Preventive Medicine, USC Keck School of Medicine, Los Angeles, CA, USA

**Keywords:** tumour microenvironment, cancer-associated fibroblast, breast cancer, glutathione S-transferase Pi, *α*-smooth muscle actin

## Abstract

**Background:**

Glutathione S-transferase Pi (GSTPi) expression is one of the factors, which is known to be associated with development of resistance to chemotherapeutics in cancer patients, including those with breast cancer. Yet, its expression has been reported to be undetectable in cancer cells in high percent of patients with primary breast cancer. However, GSTPi expression in stromal cells in breast tumour microenvironment, namely cancer-associated fibroblast (CAF), which is recognised to have major roles in cancer progression, remains poorly reported.

**Methods:**

The aim of the study was to determine the expression of GSTPi; vimetin, a fibroblast-associated cytoskeleton protein; and *α*-smooth muscle actin (*α*-SMA), a known marker of CAF in breast cancer tissue, by immunohistochemical staining method in consecutive histologic sections of formalin-fixed and paraffin-embedded tissue biopsy specimens from a cohort of 39 paired cases of patients with invasive breast cancer and the corresponding axillary lymph nodes metastases.

**Results:**

Ductal and acinar luminal epithelial cells, myoepithelial cells and surrounding fibroblasts exhibited a homogeneous cytoplasmic reactivity with anti-GSTPi antibody in 11 of 11 cases of benign breast tissue biopsies. The vimentin-positive fibroblasts were unreactive with anti-*α*-SMA antibody. Loss of GSTPi expression was observed in breast cancer cells, at both the primary and metastatic sites, in 31 of 39 paired cases, as compared with benign breast epithelial cells (Fisher's exact test *P*<0.001). A significant association was observed between GSTPi-positive, vimentin-positive and *α*-SMA-positive fibroblast in tumour microenvironment at both sites.

**Conclusion:**

This is an original report of demonstration of a significance association between tumour microenvironment-associated GSTPi-positive CAF (vimentin/*α*-SMA-positive) and the GSTPi-negative cancer cells in paired cases of primary invasive breast cancer and the corresponding axillary lymph nodes metastases.

Breast cancer is the second leading cause of death from cancer of women in the United States. Most patients with oestrogen receptor-negative breast cancer cells are treated with chemotherapeutic agents (Gonzales-Angulo *et al*, 2007). A subpopulation of such patients subsequently develop resistance to treatment, leading to the life-threatening progressive disease (Gonzales-Angulo *et al*, 2007). Role of various molecular mediators in the development of drug resistance by cancer cells have been reported. One such mediator is glutathione S-transferase Pi (GSTPi), a member of GST supergene family (Su *et al*, 2003; Arai *et al*, 2008). GSTPi catalyses reactions that result in covalent conjugation of reduced glutathione with electrophile compounds such as carcinogens and cytotoxic drugs (Brockstedt *et al*, 2002; Van Emburgh *et al*, 2008). The resulting product is hydrophilic and less toxic, and is readily excreted, thereby protecting the GSTPi protein-positive normal cells from the adverse effects of carcinogens (Sawaki *et al*, 1990). Reduction or loss of GSTPi protein expression has been reported to occur mainly by epigenetic mechanism in several forms of cancer, including breast, leading to suggestions that such loss may result in additional genetic damage in cancer cells and accelerated progression of disease (Randall *et al*, 1990; Toffoli *et al*, 1992; Green *et al*, 1993; Moskaluk *et al*, 1997; Esteller *et al*, 1998; Jhaveri and Morrow, 1998; Montironi *et al*, 1999; DeMarzo *et al*, 2003). Conversely, patients with GSTPi-positive breast cancer cells have shown to be resistant to treatment with chemotherapeutics, such as cyclophosphamide, methatrexate, adriamycin, doxorubicin, 5-fluorouracil, docetaxel or paclitaxel (Su *et al*, 2003; Arai *et al*, 2008; Yu *et al*, 2009). The results of these studies suggest that the GSTPi-positive cancer cells neutralise the cytotoxic effects of chemotherapeutic agents by a mechanism similar to that of their normal counterparts against carcinogens. Yet, GSTPi expression has been reported to be undetectable in cancer cells in high percent of cases of patients with primary invasive breast carcinoma (Esteller *et al*, 1998). These intriguing reports prompted us to identify other potential source of GSTPi expression in breast cancer tumour microenvironment-associated stroma, such as fibroblast. Such an investigation has not been reported. One of the major tumour microenvironment-associated stromal cells is referred to cancer-associated fibroblast (CAF), which has been recognised to have major roles in the progression of cancer, including that of breast cancer (Orimo *et al*, 2001; Shekhar *et al*, 2001; Desmouliere *et al*, 2004; Micke and Ostman, 2004; Nakagawa *et al*, 2004; Tang *et al*, 2004; Galiè *et al*, 2005; Micke and Ostman, 2005; Sugimoto *et al*, 2005; Cat *et al*, 2006; Ide *et al*, 2006; Patocs *et al*, 2007).

Here we present a report of a statistically significant association between the loss of GSTPi expression in breast cancer cells and the maintenance of its expression in vimentin/*α*-SMA-positive CAF in tumour microenvironment in paired cases of primary invasive breast cancer and corresponding axillary lymph node metastases.

## Materials and methods

### Reagents

Mouse monoclonal antibodies to GSTPi (clone: LW29, Isotype: IgG2a), smooth muscle actin (*α*-SMA; clone: 1A4, Isotype: IgG2a) and vimentin (clone: V9, Isotype: IgG1) were obtained from Novocastra Laboratories Ltd (Newcastle-upon-Tyne, UK); Dako Cytomation (Carpinteria, CA, USA), and Millipore (Temecula, CA, USA), respectively. Avidin–biotin–peroxidase complex (ABC), biotinylated horse antimouse immunoglobulins and normal horse serum were purchased from Vector Laboratories, Inc. (Burlington, CA, USA), and blocking peptides specific for each of the primary antibody from LabVision (Fremont, CA, USA). All other reagents used were of the highest purity available from Sigma-Aldrich Chemical Co. (St Louis, MO, USA).

### Patients

Formalin-fixed/paraffin-embedded (FFPE) archival breast tissue biopsy specimens, representing confirmed 39 paired cases of primary invasive breast cancer and corresponding axillary lymph nodes metastases were obtained from the Northern Region Hospital, Thailand. In addition, 11 cases of benign breast tissues biopsy specimens without a detectable tumour were obtained from age-matched cohort of patients as positive control tissues for the expression of GSTPi and vimentin from the same tissue bank. Moreover, one case of tissue biopsy specimen of invasive breast cancer with known expression of GSTPi and *α*-SMA was obtained as a positive control from the same tissue bank. Both the experimental and controls tissue specimens were fixed, processed and embedded in an identical manner at the above hospital. The age and pathologic parameters of patients with invasive breast cancer are shown in Table 1. Moreover, the tumour histologic grades and nuclear grades of all 39 cases of primary invasive breast cancer were independently reviewed and confirmed by three of the authors (BC, WYN and SAI). Histologic classification of the primary invasive breast cancer was determined according to the criteria of Scarff–Bloom–Richardson (Bloom and Richardson, 1957; Scarff and Torloni, 1968). The use of tissue biopsy specimens for this study was approved by the institutional review board, Chiang Mai University, Thailand, in accordance with the ethical policy and procedure.

### Immunohistochemical staining

The tissue blocks were sectioned at 4 *μ*m thickness, placed on histologic glass slide and subjected to immunostaining as previously described (Imam *et al*, 1993). Briefly, unstained sections on histologic glass slides were deparaffinized in xylene and rehydrated in decreasing concentration of alcohol. Endogenous peroxidase activity was quenched with hydrogen peroxide (H_2_O_2_) in methanol for 20 min. The unmasking of epitopes of the antigens was performed by heating the slides in 0.01 M sodium citrate buffer, pH 6.0, in a microwave pressure cooker for 30 min. Following the incubation of slides with normal appropriate serum for 20 min to block nonspecific binding of the subsequent antibodies, sections were incubated overnight with 150 *μ*l of mouse monoclonal antibody to GSTPi (0.4 *μ*g ml^−1^), *α*-SMA (0.14 *μ*g ml^−1^) or vimentin (0.08 *μ*g ml^−1^) in a humidified chamber. The optimum concentration of each primary antibody was empirically determined to yield the most intense immunostaining of known target cell in benign breast or breast cancer tissue sections with an undetectable or negligible staining of nonspecific cells and stromal component. Biotinylated horse anti-mouse immunoglobulins antibodies as secondary antibody, followed by ABC conjugate were applied to the sections. The concentrations of the secondary antibody and the ABC reagents used were as previously determined to be optimum on FFPE tissue biopsy specimens of breast cancer (Saha *et al*, 2007). Diaminobenzidine was used as the chromogen and haematoxylin as the counterstain. For each experiment, a control that consisted of the pre-absorbed primary monoclonal antibody with the specific blocking peptide (10 mg protein per ml of working dilution of the antibody) was included to determine specificity of the antibody reaction.

### Evaluation of immunohistochemical staining

**Figure 1:**
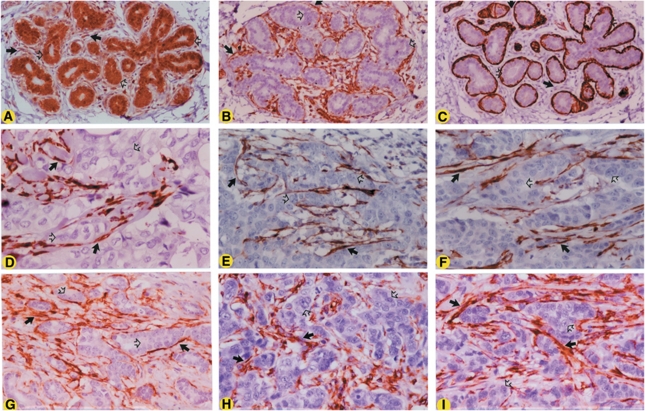
Immunohistochemical localisation of GSTPi, vimentin or *α*-SMA in benign breast and breast cancer tissue biopsy specimens. Sections (4 *μ*m thick) of FFPE biopsy specimens of benign breast and a paired case of primary invasive breast cancer and corresponding metastasis to axillary lymph node were immunohistochemically stained with mouse monoclonal antibody to GSTPi, vimentin or *α*-SMA. The anti-GSTPi antibody exhibited homogeneous cytoplasmic brown staining of the benign breast ductal and acini luminal epithelial cells, myoepithelial cells and surrounding fibroblasts (brown staining indicated by opened arrows for epithelial/myoepithelial cells and closed arrows for fibroblasts, **A**). Anti-vimentin antibody showed a homogeneous cytoplasmic brown staining of fibroblasts (indicated by closed arrows, **B**), but not with epithelial cells (indicated by opened arrows, **B**). Anti-*α*-SMA antibody exhibited no detectable reactivity with surrounding fibroblasts (indicated by closed arrows, **C**), and luminal epithelial cells (indicated by opened arrows, **C**), whereas homogeneous reactivity was observed with myoepithelial cells in benign breast (indicated by brown staining of outmost layer of cells, **C**). Both primary and axillary lymph node metastatic breast cancer cells showed no detectable reactivity with anti-GSTPi antibody (absence of brown staining indicated by opened arrows; **D** and **G**, respectively), whereas the tumour microenvironment-derived fibroblasts exhibited homogeneous reactivity (brown staining indicated by closed arrows; **D** and **G**, respectively). Anti-vimentin antibody exhibited homogeneous cytoplasmic brown staining of tumour microenvironment-derived fibroblasts at both primary and lymph node metastasis sites (indicated by closed arrows; **E** and **H**, respectively), but not with the cancer cells (absence of brown staining indicated by opened arrow; **E** and **H**, respectively). A similar pattern of reactivity of anti-SMA antibody was observed with fibroblast at both the primary (indicated by closed arrows, **F**) and lymph node metastasis site (indicated by closed arrow, **I**), whereas the cancer cells were negative (indicated by opened arrows, **F** and **I**). The sections were counterstained with Harris’ s hematoxylin (blue nuclear staining). Original magnification, × 150 (**A**–**C**) and × 312 (**D**–**I**).

Three of the authors (BC, WYN and SAI) independently reviewed the immunostained tissue sections in a fully blinded manner. The data from immunostaining analysis were stratified into three categories, based on the frequency of expression of GSTPi, *α*-SMA or vimentin, namely, homogeneous with most cells showing positivity (a pattern similar to that of the benign breast epithelial cells as illustrated in Figures 1A–C), heterogeneous with a few scattered patches of cells with positivity or negative (less than 2% of cells with positivity) as described elsewhere (Saha *et al*, 2007).

### Statistical analysis

Expression of GSTPi, *α*-SMA or vimentin was grouped as homogeneously positive, heterogeneously positive or negative as described above. Contingency tables and the Fisher's exact test (Mehta and Patel, 1993) were used to summarise the association between the protein analytes and cell types.

## Results

### Benign breast

Monoclonal anti-GSTPi antibody showed homogeneous reactivity with the cytoplasm/nuclear of ductal and acinar luminal epithelial cells and myoepithelial (basal) cells in 11 of 11 cases of benign breast tissue specimens (Figure 1A, Table 2). Moreover, a homogeneous cytoplasmic/nuclear expression of GSTPi was observed in stromal fibroblasts in 10 of 11 (91%) cases, but was undetectable in one case, although breast ductal, acinar and myoepithelial cells were positive. Monoclonal anti-vimentin antibody gave a homogeneous cytoplasmic pattern of reactivity in stromal fibroblasts in 11 of 11 cases (Figure 1B; Tables 2 and 3). The benign ductal and acinar epithelial cells and myoepithelial cells were negative for the expression of vimentin (Figure 1B). Anti-*α*-SMA antibody exhibited a homogeneous cytoplasmic reactivity with myoepithelial cells, whereas ductal and acinar luminal epithelial cells and stromal fibroblasts were negative in 11 of 11 cases (Figure 1C; Table 2).

### Primary and metastatic breast cancer

Breast cancer cells in paired primary tumours and their respective metastases to axillary lymph node exhibited homogeneous cytoplasmic/nuclear expression of GSTPi in 8 of 39 (21%) and heterogeneous expression in 1 of 39 (3%), whereas the 29 of 39 (74%) cases were negative at both primary and metastatic sites (Figures 1D and G; Table 2). In the remaining one paired case, the cancer cells at the primary site were heterogeneously positive for the expression of GSTPi, whereas those at the metastatic site were negative (Table 2). A highly significant loss of GSTPi expression was detected in breast cancer cells, both at the primary and metastatic sites, as compared with benign breast epithelial cells (Fisher's exact test *P*<0.001, Table 2).

### Tumour microenvironment

The tumour microenvironment-associated stromal fibroblasts exhibited a homogeneous expression of vimentin at both the primary and metastatic sites in 38 of 39 (97%) paired cases (Figures 1E and H; Table 2). In the remaining one paired case, the fibroblasts were homogeneously positive for the expression of vimentin at the primary site, whereas those at the metastatic site were heterogeneously positive (Table 2). The fibroblasts in the tumour microenvironment, at both the primary and metastatic sites, exhibited homogeneous expression of *α*-SMA in 31 of 39 (79%) paired cases (Figures 1F and I; Table 2), demonstrating these cells being CAF. Fibroblasts with heterogeneous expression of *α*-SMA-positive fibroblasts were observed at both sites in one paired case and at the primary site in five cases (Table 2). The *α*-SMA-negative fibroblasts were detected at both sites in two paired cases and at the metastatic site in one case (Table 2). A statistically significant number of cases exhibited the expression of *α*-SMA in tumour microenvironment-associated stromal fibroblasts, both at the primary and metastatic sites, as compared with benign breast stromal fibroblasts (Fisher's exact test *P*<0.001, Table 2). Moreover, a significant association was also observed between vimentin-positive and *α*-SMA-positive fibroblasts or CAF in tumour microenvironment in 37 of 39 (95%) cases of primary breast cancer and in 36 of 39 (92%) cases of lymph node metastases (Table 3).

Tumour microenvironment-associated stromal fibroblasts exhibited homogeneous expression of GSTPi at both the primary and metastatic sites in 30 of 39 (77%) (Figures 1D and G) and heterogeneous pattern in 3 of 39 (8%) paired cases (Table 2). The remaining paired cases showed mixed patterns of staining, with two being negative at both sites and two at the primary site (Table 2). A highly significant association was observed between GSTPi-positive/vimentin-positive/*α*-SMA-positive tumour microenvironment-associated stromal fibroblast or CAF at both the primary and metastatic sites in this cohort of patients (Fisher's exact test *P*<0.001, Table 2). No significant association was observed between GSTPi-positive/vimentin-positive/*α*-SMA-positive CAF and patients’ age at diagnosis, tumour histologic grade, tumour nuclear grade, or number of lymph nodes involved.

### Controls

The application of pre-absorbed antibody to GSTPi, vimentin or *α*-SMA with appropriate blocking peptide led to the abolition of immunostaining of the specific target cells, which exhibited reactivity with that of the unabsorbed counterpart, demonstrating the specificity of reactivity of the antibodies. Monoclonal antibodies to GSTPi or vimentin consistently exhibited reactivity with the target cells in tissue sections from the known positive control of benign breast tissue, whereas anti-*α*-SMA antibody was non-reactive (results now shown). Moreover, monoclonal antibodies to GSTPi or *α*-SMA showed reactivity with the target cells in tissue sections from the known positive control of invasive breast cancer tissue (results not shown).

## Discussion

GSTPi expression, one of the known factors which is associated with the development of resistance to chemotherapeutics in cancer patients, including those with invasive breast cancer (Su *et al*, 2003; Arai *et al*, 2008; Yu *et al*, 2009), is widely reported to be downregulated in cancer cells in high percent of cases of patients with invasive breast cancer (Esteller *et al*, 1998). These intriguing reports prompted us to investigate the expression of GSTPi in breast tumour microenvironment-associated stromal fibroblast in particular, given their recognised roles in cancer progression (Orimo *et al*, 2001; Shekhar *et al*, 2001; Desmouliere *et al*, 2004; Micke and Ostman, 2004; Nakagawa *et al*, 2004; Tang *et al*, 2004; Galiè *et al*, 2005; Micke and Ostman, 2005; Sugimoto *et al*, 2005; Cat *et al*, 2006; Ide *et al*, 2006; Patocs *et al*, 2007).

The focus of this study was to determine GSTPi expression in invasive breast cancer cells and breast tumour microenvironment-associated stromal cells and the latter's cellular identity by immunohistochemical staining method in consecutive sections of paired tissue biopsy specimens, from a well-characterized cohort of 39 patients with primary invasive breast cancer and corresponding axillary lymph nodes metastasis. Sections of benign breast tissue specimens without any detectable cancer cells were utilised as positive controls for the expression of GSTPi and vimentin, and negative for *α*-SMA expression. Vimentin-positive fibroblasts in benign breast tissue sections were consistently negative for the expression of *α*-SMA, a known marker of CAF. Moreover, GSTPi was found to be homogeneously and consistently expressed in luminal epithelial and myoepithelial cells in benign breast ducts and acini. These results are in agreement with those reported by others (Terrier *et al*, 1990). The observation suggests that both luminal epithelial and myoepithelial cells with homogeneous expression of GSTPi may contribute to protecting normal or benign breast tissue from the adverse affects of environmental pollutants and carcinogens.

GSTPi-positive breast cancer cells were detected at both the primary and metastatic sites in nine paired cases. This subgroup of patients with continued expression of GSTPi in cancer cells is likely to contribute to the development of resistance to chemotherapeutic agents by a mechanism which could be similar to that utilised by normal cells to neutralise cytotoxic effects of environmental carcinogens (Brockstedt *et al*, 2002; Su *et al*, 2003; Arai *et al*, 2008). Conversely, breast cancer cells that were negative for the expression of GSTPi at both the primary and the metastatic sites were found in 29 of 39 (74%) paired cases. A statistically significant association was observed between patients with GSTPi-negative breast cancer cells and GSTPi-positive CAF, at both the primary and metastatic sites, with 95% confidence intervals (Table 2). The association is large enough that it cannot likely be explained by random chance alone. That is, the calculation of a *P*-value incorporates the sample size, as well as the magnitude of the association. This subgroup of patients with GSTPi-negative cancer cells, in principle, might be expected to respond to the application of chemotherapeutic agents. However, GSTPi-negative cancer cells may escape from the cytotoxic effects of chemotherapeutic agents by neutralising them through the release of GSTPi from the omnipresent GSTPi-positive CAF in the tumour microenvironment as discussed below.

In contrast to benign breast tissue, a statistically significant association was observed between vimentin-positive and *α*-SMA-positive fibroblasts in the tumour microenvironment at both the primary and metastatic sites. Overexpression of *α*-SMA in fibroblasts has been reported to be one of the phenotypic characteristics that identifies these cells as CAF (Tuxhorn *et al*, 2002; Mishra *et al*, 2008). In the present study, CAF were positive for the expression of GSTPi in a highly significant number of cases. These GSTPi-positive CAFs in the tumour microenvironment may contribute to protecting the GSTPi-negative cancer cells by catalysing GSTPi-mediated reactions that would neutralise chemotherapeutic agents in this subgroup of patients. Such cancer cells, in the absence of GSTPi-positive CAF in tumour microenvironment, would otherwise be susceptible to the cytotoxic effects of chemotherapeutic agents. Indeed, recent studies have reported on the roles of bone marrow-derived stromal cells in protecting myeloproliferative neoplasms or acute myeloid leukaemia from the effects of JAK2 or FLT3 tyrosine kinase inhibitors, respectively (Manshouri *et al*, 2011; Parmar *et al*, 2011). These studies clearly show the role of tumour microenvironment-associated cells in protecting cancer cells from the intended effects of treatment.

It is recognised that, in addition to GSTPi, other mediators of multidrug resistance are also involved in the development of chemoresistance in patients with breast carcinoma (Morrow *et al*, 1998). However, the demonstration in the present study of overexpression of GSTPi in *α*-SMA-positive CAF is new and raises the possibility that their presence in tumour microenvironment could be a contributing factor that may act alone or in synergy with other factors for the development of chemoresistance in patients with GSTPi-negative breast cancer cells. Hence, an approach to target simultaneously both the GSTPi-positive cancer cells and the GSTPi-positive CAF in the tumour microenvironment holds promise as a more effective approach to overcoming chemoresistance in individual patients with breast cancer.

Owing to the absence of information about the clinical follow-up, with respect to recurrent or development of resistance to chemotherapeutics in this cohort of patients, GSTPi expression in CAF could not be evaluated as a potential prognostic marker. In this respect, a follow-up study is being organised to retrospectively determine the efficacy of GSTPi expression in CAF as a potential prognostic marker for the development of chemoresistance in a cohort of larger numbers of patients with invasive breast cancer.

## Figures and Tables

**Table 1:** Pathological characteristics and age of patients with metastatic breast cancer

**Factors**	**No. of patients**	**Percent (%)**
Total no. of patients	39	100
		
*Age at diagnosis (years)*		
<50	20	51
⩾50	19	49
Median (range)	49 (34–77)	
		
*Tumor histologic grade*		
1	0	0
2	32	82
3	7	18
		
*Tumor nuclear grade*		
1	0	0
2	32	82
3	7	18
		
*Lymph nodes positive (number)*		
⩽5	21	54
>5	18	46
Median (range)	5 (2–28)	

**Table 2:** Comparison of association between immunohistochemical expression of GSTPi, vimentin or *α*-SMA in benign breast and paired cases of primary breast cancer or metastasis to axillary lymph nodes

**Markers expression**	**Benign breast (*n*=11)**	**Primary breast cancer (*n*=39)**	**LN metastasis (*n*=39)**	***P*-value^a^**	***P*-value^b^**
*GSTPi expression in breast cells*				<0.001	<0.001
Negative (%)	0 (0) (0, 26)^c^	29 (74) (58, 86)^c^	30 (77) (60%, 88)^c^		
Heterogeneous (%)	0 (0)	2 (5)	1 (3)		
Homogeneous (%)	11 (100)	8 (21)	8 (21)		
					
*GSTPi expression in fibroblast*				1.00	0.33
Negative (%)	1 (9) (0, 40)^c^	4 (10) (3, 25)^c^	2 (5) (1, 19)^c^		
Heterogeneous (%)	0 (0)	3 (8)	7 (18)		
Homogeneous (%)	10 (91)	32 (82)	30 (77)		
					
*Vimentin expression in fibroblast*				NA	1.00
Negative (%)	0 (0) (0, 26)^c^	0 (0) (0, 11)^c^	0 (0) (0, 11)^c^		
Heterogeneous (%)	0 (0)	0 (0)	1 (3)		
Homogeneous (%)	11 (100)	39 (100)	38 (97)		
					
*Alpha-SMA expression in fibroblast*				<0.001	<0.001
Negative (%)	11 (100) (74, 100)^c^	2 (5) (1, 19)^c^	3 (8) (2, 22)^c^		
Heterogeneous (%)	0 (0)	6 (15)	1 (3)		
Homogeneous (%)	0 (0)	31 (79)	35 (90)		

Abbreviations: GSTPi=glutathione S-transferase Pi; *α*-SMA=*α*-smooth muscle actin; NA=not available.

a *P*-value based on Fisher's exact test, comparing benign breast with primary beast cancer.

b *P*-value based on Fisher's exact test, comparing benign breast with breast cancer metastasis to lymph node.

c 95% confidence intervals.

**Table 3:** Comparison of association between immunohistochemical expression of vimentin and *α*-SMA in benign breast and paired cases of primary invasive breast cancer or metastasis to axillary lymph node

	**Vimentin-positive fibroblast**			
***α*-SMA-positive fibroblast**	**Negative**	**Heterogeneous**	**Homogeneous**	**Association of expression (%)^a^**
*Benign breast*				0
Negative	0	0	11	
Heterogeneous	0	0	0	
Homogeneous	0	0	0	
				
*Primary breast cancer*				95
Negative	0	0	2	
Heterogeneous	0	0	6	
Homogeneous	0	0	31	
				
*Breast cancer metastasis to LN*				92
Negative	0	1	2	
Heterogeneous	0	0	1	
Homogeneous	0	0	35	

Abbreviation: *α*-SMA=*α*-smooth muscle actin.

a Association between the expression of vimentin and *α*-SMA in benign breast and paired cases of primary invasive breast cancer or metastasis to lymph nod.
